# Semantics vs. statistics in chemical markup

**DOI:** 10.1186/1758-2946-4-S1-O16

**Published:** 2012-05-01

**Authors:** Colin Batchelor

**Affiliations:** 1 Royal Society of Chemistry, Thomas Graham House, Cambridge, CB4 0WF, UK

## 

Since the late 1990s, natural language processing (NLP) has seen a massive shift from high-precision, low-recall systems based on small sets of hand-written rules, to methods based on the statistical analysis of large corpora. The field of chemoinformatics, likewise, is dominated by statistical and machine-learning approaches. In recent years, however, pharmaceutical companies have been engaging more and more with Semantic Web technologies, which are largely built around the sorts of hand-written systems that NLP has moved away from this century. We discuss where our current text analysis and Semantic Web efforts at the Royal Society of Chemistry are headed and how we're making use of the unreasonable effectiveness of data.

